# Efficiency of Text Message Contact on Medical Safety in Outpatient Surgery: Retrospective Study

**DOI:** 10.2196/14346

**Published:** 2020-09-10

**Authors:** Jeremy Peuchot, Etienne Allard, Bertrand Dureuil, Benoit Veber, Vincent Compère

**Affiliations:** 1 Department of Anesthesiology and Critical Care Rouen University Hospital Rouen France; 2 Le Havre Hospital Le Havre France; 3 Day Surgery Unit Rouen University Hospital France; 4 Normandie University Institut National de la Santé et de la Recherche Médicale Unité 982 Rouen France

**Keywords:** outpatient surgery, short message service (SMS), patient information, organizational, cost, unanticipated admission, preoperative instructions

## Abstract

**Background:**

Establishing pre- and postoperative contact with patients is part of successful medical management in outpatient surgery. In France, this is mostly done via telephone. Automated information with short message service (SMS) reminders might be an interesting alternative to increase the rate of compliance with preoperative instructions, but no study has shown the safety of this approach.

**Objective:**

The objective of this study was to evaluate the impact of pre- and postoperative automated information with SMS reminders on medical safety in outpatient surgery.

**Methods:**

We conducted a retrospective, single-center, nonrandomized, controlled study with a before-after design. All adult patients who had outpatient surgery between September 2016 and December 2017 in our university hospital center were included. Before April 2017, patients were contacted by telephone by an outpatient surgery nurse. After April 2017, patients were contacted by SMS reminder. All patients were contacted the day before and the day after surgery. Patients contacted by SMS reminder were also contacted on day 7 after surgery. The primary end point was the conversion rate to full-time hospitalization. Secondary end points were hospitalization causes (anesthetic, surgical, organizational) and hospitalization costs.

**Results:**

A total of 4388 patients were included, 2160 before and 2228 after the introduction of SMS reminders. The conversion rate to full-time hospitalization was 34/4388 (0.77%) with a difference between SMS group (8/2228, 0.36%) and telephone group (26/2160, 1.20%). The cost of SMS reminders was estimated as half that of telephone calls.

**Conclusions:**

In this work, we report a decrease in the rate of conversion to full-time hospitalization with the use of pre- and postoperative SMS reminders. This new approach could represent a safe and cost-effective method in an outpatient surgery setting.

## Introduction

Outpatient surgery represents a major challenge in the organization of care. The increasing number of outpatient surgeries highlights the need to ensure the highest level of safety for each patient. Establishing contact with patients before and after surgery is part of successful medical management in outpatient surgery, and it is strongly recommended by several practice guidelines [1,2].

Until recently, outpatients in France were mainly contacted by telephone before their surgery [3]. Telephone contact is costly and time-consuming with no guarantee of actually reaching the patient. According to Gaucher et al [4], the rate of telephone call failure to contact outpatients was 20.82% (425/2041 patients), whereas everyone can receive a short message service (SMS) text message so long as their mobile telephone is switched on. The use of SMS to contact surgery outpatients seems an interesting alternative to the telephone and has already been studied in chronic diseases and adherence to long-term treatments.

Several studies have shown the benefit of using SMS reminders [5,6] in patients with high blood pressure to decrease systolic blood pressure compared with usual care at 12 months or improve medication adherence. Others have developed a bank of text messages for the prevention of recurrent cardiovascular events [7]. A bank of 137 mobile telephone text messages designed to support behavior change and decrease cardiovascular risk has been developed through a multistep iterative process. During the testing of those 137 text messages, 92% of participants found the messages easy to understand and 86% found the messages contained useful information.

SMS reminders and a smartphone app have been used successfully to monitor and reduce the alcohol consumption of military veterans from a median of 5.6 units per drinking day in the first week to 4.7 units by the last week during the 4 weeks of study [8]. In contrast, inconsistent results were found in suicide prevention. There was no significant effect on likelihood or severity of current suicidal ideation or likelihood of a suicide risk incident or on emergency department visits [9].

Patient safety is a major concern for all medical teams. In studies, safety is defined as the occurrence of perioperative complications, conversion to full-time hospitalization, or rehospitalization after outpatient surgery. The most frequently used indicator is the conversion rate to full-time hospitalization [10-13]. Previous studies have shown that the use of SMS reminders before outpatient surgery increased the rates of compliance with preoperative instructions [14] and reduced the number of cancellations in gastrointestinal endoscopy [15]. The objective of this study was to evaluate the impact of pre- and postoperative SMS messages on medical safety in outpatient surgery.

## Methods

### Study Design

This was a retrospective, single-center, nonrandomized, controlled study with a before-after design. All data were extracted retrospectively from the database about patients who were hospitalized in the adult outpatient surgery unit of a French university hospital center from September 2016 to December 2017. Two groups of patients were formed corresponding to two study periods: September 2016 to March 2017 and April 2017 to December 2017. Ethical approval for this study (No. E2019-11) was provided by the noninterventional research committee based at Rouen University Hospital, Rouen, France. The requirement for written informed consent was waived by the committee.

### Patient Selection

Patients scheduled for orthopedic; ear, nose, and throat; oral; dental; gynecologic; vascular; and thoracic outpatient surgery were included. Patients were eligible if they were aged over 18 years and had a mobile telephone. Patients were excluded if they were pregnant, younger than age 18 years, or did not speak French [1].

### Study Procedure

**Figure 1:**
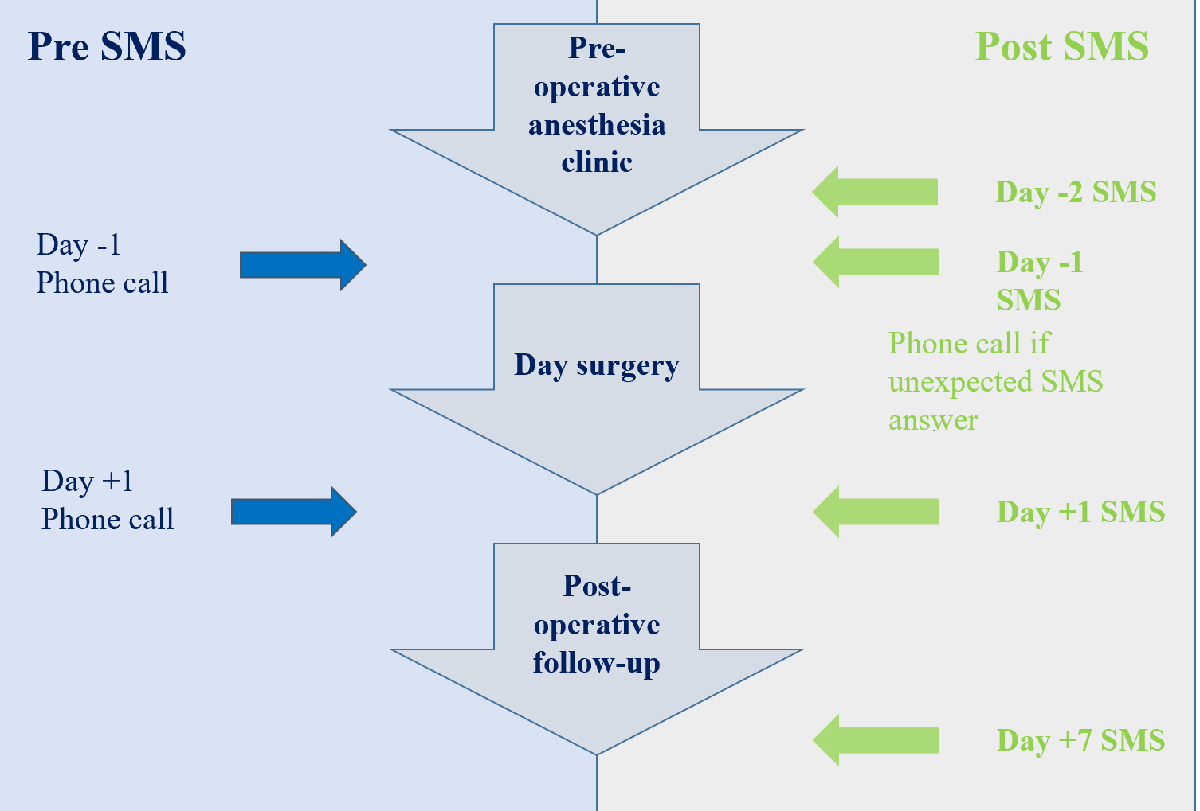
Course in outpatient surgery before and after short message service text reminders.

Before April 2017, the first group of patients (telephone group) was contacted by telephone the day before and the day after surgery by an outpatient surgery nurse (Figure 1). After April 2017, the second group of patients (SMS group) was contacted by text message the day before surgery and on day 1 and day 7 after surgery (Figure 1). The deployment of a new version of scheduling software allowed us to send an SMS reminder to all patients with scheduled outpatient surgery. As soon as the outpatient surgery was scheduled, a nurse was able to set up SMS reminders, which were automatically sent. Patients’ mobile telephone numbers are stored in the Gestime software internally developed by our university hospital center and connected to Computerized Medical Records CDP (GIP Cpage).

**Figure 2:**
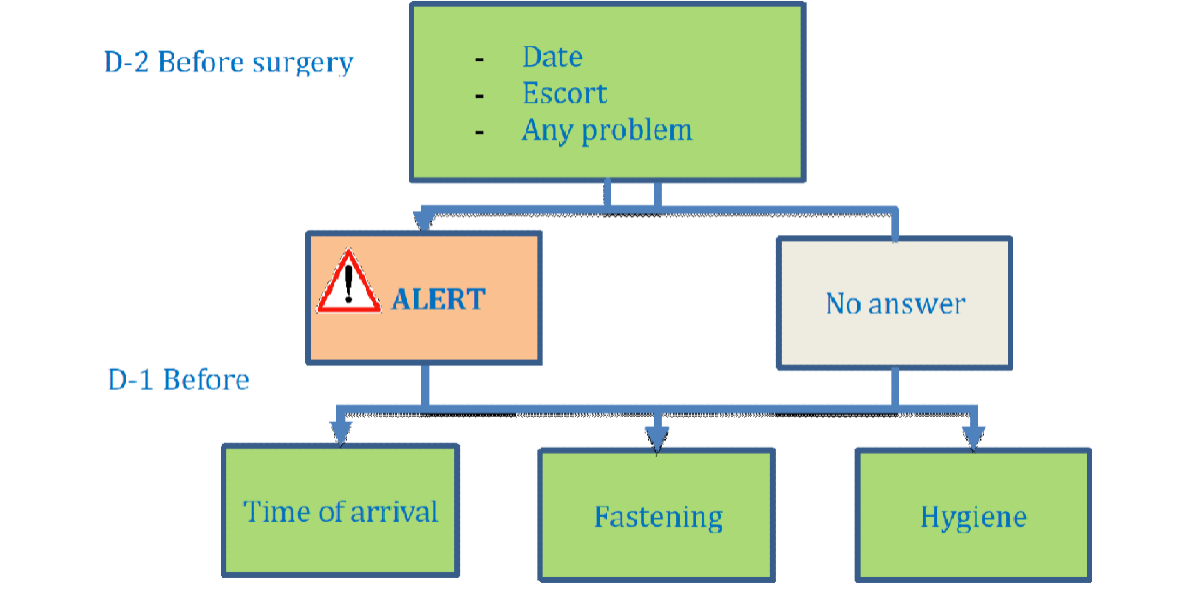
Short message service text pattern before surgery.

Messaging started two days before surgery with an SMS reminder and the possibility of alerting the medical staff if the patient was unable to attend surgery. The patient was able to respond ALERT if there was a medical problem or if assistance was required. A response different to ALERT was categorized as an unexpected response. There was no obligation to respond to the SMS reminder (Figure 2). We noted the percentage of patients who received the message but did not have the ability to know if messages were read or not. The day before surgery, the patient received 3 messages informing them of the required time of arrival and location of the outpatient unit, fasting recommendations, and hygiene rules (Figure 2).

**Figure 3:**
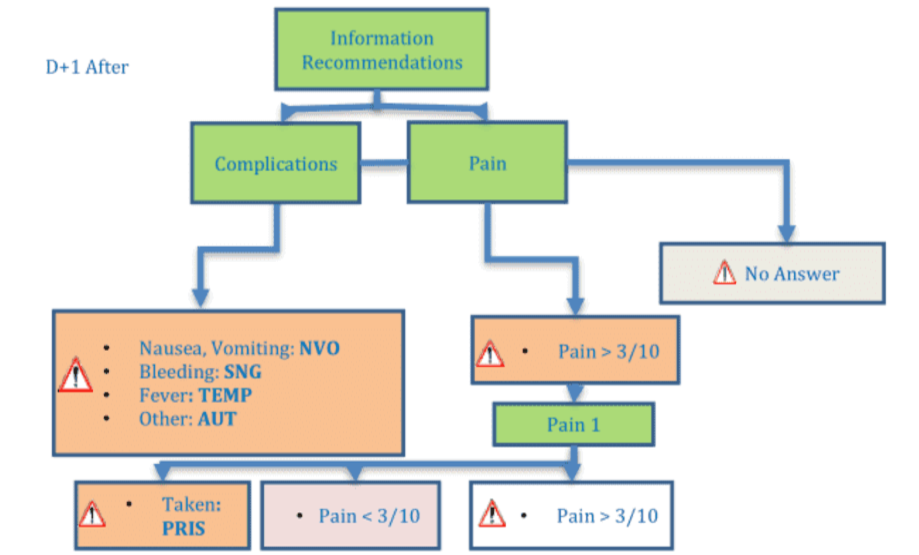
Short message service text pattern day 1 after surgery.

After surgery, patients received several SMS reminders with the possibility to answer. On day 1 after surgery, patients received an SMS reminder with recommendations on pain medication, a question about the onset of complications (nausea, vomiting, bleeding, fever, or other), and a pain evaluation on a numerical scale from 0 to 10 (Figure 3). If patients rated their pain more than 3/10, a reminder was sent to take their pain medication or immediately answer (TAKEN) if their pain medication had been taken. Patients were asked to send another pain evaluation 1 hour after taking their pain medication.

**Figure 4:**
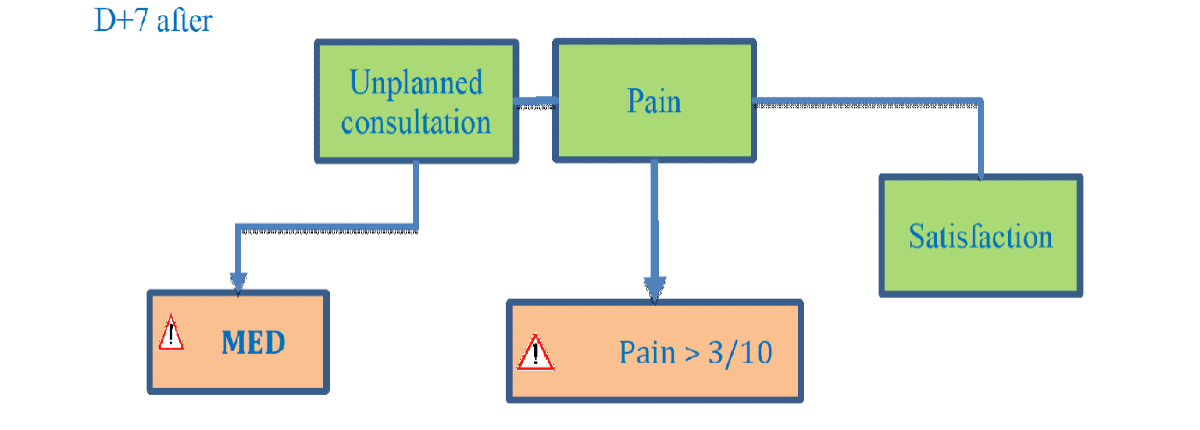
Short message service text pattern day 7 after surgery.

On day 7 after surgery, patients received one SMS reminder asking if they had had an unplanned consultation since their surgery and a second one asking them to evaluate their pain (Figure 4). Patients received one last message asking them to rate their global satisfaction with their outpatient care using a numerical scale from 0 to 10. Examples of SMS reminders are shown in Multimedia Appendix 1.

### Data Collection

Data were extracted from the medical computerized database of our university hospital center. For hospitalized patients, anesthesia and surgery charts were reviewed. Perioperative data were collected.

- Preoperative: age, sex, BMI, comorbidities, allergies, smoking status, American Society of Anesthesiologists physical status classification (an assessment of the patient’s preanesthesia medical comorbidities)
- Perioperative: type of surgery, type of anesthesia, length of surgery, time of end of surgery
- Postoperative: length of stay in postanesthesia care unit, pain evaluation using a 100-mm horizontal visual analog scale, need for complementary morphine titration, postoperative nausea and vomiting

SMS data were extracted from the computerized MemoQuest (Calmedica) database.

### Outcome Variables

The primary end point was the conversion rate to full-time hospitalization. Secondary end points included cancellation rates on the day of surgery and a medicoeconomic study of SMS reminder use. In addition, causes of conversion to full-time hospitalization (anesthetic: persisting pain, postoperative nausea and vomiting, sickness, aspiration syndrome; surgical: difficulty or length more than 2 hours; and organizational: entering or exiting operating room after 3:00 pm) and preoperative message (rate of ALERT replies, unexpected replies, undelivered SMS reminders) and postoperative pain (pain evaluation on day 1) data were collected. Last, data on pain evaluation and satisfaction and declarative rate of rehospitalization or unplanned medical consultation on day 7 postoperatively were collected, but we could not examine links between these different items.

### Statistical Analysis

This study investigating the safety of SMS reminder contact before outpatient surgery did not require an a priori calculation of the number of subjects required. The values are presented as absolute and percentage for categorical variables and as mean and standard deviation for quantitative variables. The quantitative variables were compared using a Student *t* test (if the distribution was normal) or a Mann-Whitney *U* test (if the distribution was not normal). The categorical variables were analyzed using a Fisher or chi-square test. The significance threshold was set at .05. All statistics were produced using Prism 5.0 (GraphPad Software).

## Results

### Patient Characteristics

Between September 2016 and December 2017, 4388 patients were included in the study, 2160 in the telephone group and 2228 in the SMS group. Patient characteristics are presented in Table 1.

**Table 1:** Patient characteristics (n=4388).

Characteristic		Total (n=4388)	Telephone group (n=2160)	SMS^a^ group (n=2228)	*P* value
Age in years, mean (SD)		43.2 (17.6)	43.0 (17.4)	43.3 (17.9)	.61
**Sex, n (%)**					
	Women	3011 (68.62)	1466 (68.87)	1545 (69.34)	.67
	Men	1377 (31.38)	694 (32.13)	683 (30.66)	.47
Length of surgery (min), mean (SD)		51.5 (32.6)	52.5 (30.6)	50.5 (31.6)	.04
**Type of surgery, n (%)**					
	Thoracic	198 (4.51)	76 (3.52)	122 (5.48)	.002
	Vascular	136 (3.10)	64 (2.96)	72 (3.23)	.67
	Oral	296 (6.75)	168 (7.78)	128 (5.75)	.009
	Dental	190 (4.33)	87 (4.03)	103 (4.62)	.37
	Ear, nose, and throat	417 (9.50)	225 (10.42)	192 (8.62)	.04
	Orthopedic	1578 (35.96)	817 (37.82)	761 (34.16)	.01
	Gynecologic	1573 (35.85)	723 (33.47)	850 (38.15)	.001

^a^SMS: short message service.

### Primary and Secondary Outcomes

The overall conversion rate to full-time hospitalization was 0.78% (34/4388); 1.20% (26/2160) in the telephone group versus 0.36% (8/2228) in the SMS group (*P*=.001). The conversion rates to full-time hospitalization and cause of hospitalization are shown in Table 2.

There was a significantly higher rate of cancellations declared on the day of surgery in the SMS group than in the telephone group: 3.66% (79/2160) versus 2.24% (50/2228); *P*=.02. There was no statistically significant difference in the demographic characteristics of patients converted to full-time hospitalization between groups (Table 3). Most of the surgeries were done under general anesthesia (25/34, 74%). Among all surgeries, orthopedic surgery accounted for 50% (17/34) and gynecologic surgery 44% (15/34).

In our study, 95.60% (2130/2228) of patients contacted by SMS reminder after their surgery actually received the reminder. For SMS reminder 1 sent on day 2 before surgery, 0.81% (18/2228) of patients replied ALERT and 6.24% (139/2228) sent an unexpected response. Most of the unexpected responses confirmed the patient’s attendance at surgery.

For SMS reminder 2 sent on day 1 after surgery, 7.00% (156/2228) of patients rated their pain as more than 3/10. For SMS reminder 3 sent on day 7 after surgery, 2.60% (58/2228) of patients rated their pain as more than 3/10, and 6.69% (149/2228) of patients replied that they had seen their general practitioner or had been rehospitalized. A total of 90.98% (2027/2228) of patients gave a satisfaction score of 7/10 or more. The mean satisfaction score was 8.7 (SD 1.9).

**Table 2:** Conversion rates to full-time hospitalization between groups and according to the cause of hospitalization (n=4388).

Cause	Telephone group (n=2160)	SMS^a^ group (n=2228)	*P* value
Total	26 (1.20)	8 (0.36)	.001
Anesthetic	15 (0.69)	4 (0.18)	.01
Surgical	7 (0.32)	3 (0.13)	.22
Organizational	4 (0.19)	1 (0.04)	.12

^a^SMS: short message service.

**Table 3:** Characteristics of patients converted to full-time hospitalization.

Variable		Telephone group (n=26)	SMS^a^ group (n=8)	Total (n=34)
**Type of anesthesia, n (%)**				
	General	20 (77)	5 (63)	25 (74)
	General + regional	4 (15)	1 (13)	5 (15)
	Regional	1 (4)	1 (13)	2 (6)
	Spinal	1 (4)	1 (13)	2 (6)
**Type of surgery, n (%)**				
	Orthopedic	12 (46)	5 (63)	17 (50)
	Gynecologic	12 (46)	3 (38)	15 (44)
	Oral	2 (8)	0 (0)	2 (6)
Length of surgery (min), mean (SD)		84.4 (60.7)	88.7 (58.1)	85.5 (58.6)
Length of stay in PACU^b^ (min), mean (SD)		87.5 (37.1)	75 (58.6)	84.6 (50.5)
VAS^c^, mean (SD)		3.3 (3.3)	1.7 (2.9)	2.9 (3.1)
Morphine titration, n (%)		4 (15)	2 (25)	6 (18)
Morphine dose (mg), mean (SD)		1.8 (4.2)	1.7 (3.2)	1.7 (3.7)
Prevention of PONV^d^, n (%)		22 (85)	7 (88)	29 (85)
PONV, n (%)		24 (92)	8 (100)	32 (94)
Length of hospitalization in days, mean (SD)		3.0 (10.4)	2.5 (3.9)	2.9 (8.5)

^a^SMS: short message service.

^b^PACU: postanesthesia care unit.

^c^VAS: visual analog scale.

^d^PONV: postoperative nausea and vomiting.

### Economic Aspect of Short Message Service Versus Telephone

Until recently, outpatients were mainly contacted by telephone by an outpatient surgery nurse before their surgery. However, telephone contact is time-consuming, representing 15 minutes per patient. In our study, there were approximately 3600 outpatients per year. At 15 minutes per patient, around 900 hours per year are spent contacting patients by telephone. Considering that the annual work time of a full-time nurse in France is 1607 hours [16], based on our findings, 56% of a nurse’s time is spent contacting patients by telephone. Given that the annual cost of a full-time nurse is estimated at around €40,000 (US $45,018), the time spent contacting patients by telephone represents an overall cost of €22,400 (US $25,210) and an estimated cost of €6 (US $6.75) per patient. The use of an SMS reminder platform like MemoQuest (Calmedica), at an estimated cost of €3 (US $3.37) per patient [17], represents an annual cost of €10,800 (US $12,154) for 3600 patients. The cost of an SMS reminder was estimated as half that of a telephone call. The nurse time to check the SMS platform is negligible. One patient a day is called back after SMS reminder responses. The duration of the contact is much shorter (5 minutes) because the problem has already been focused by the SMS reminder.

## Discussion

### Principal Findings

In this work, we report a decrease in the rate of conversion to full-time hospitalization with the use of pre- and postoperative SMS reminders in an outpatient surgery setting. The rate of conversion to full-time hospitalization is one of the main criteria for monitoring safety in outpatient surgery [18]. We found a lower rate of conversion (1.2%) compared with other studies. Cousin et al [19] in 2012 observed a rate of 3.1% in a retrospective study including 15,994 patients. Whippey et al [20], in a case-control study with 20,657 patients, observed a 2.7% conversion rate. Fortier et al [12], in a prospective study published in 1998 including 15,172 patients, reported a hospitalization rate of 1.4%.

Several hypotheses may underlie this decrease in the rate of conversion after the implementation of SMS reminders. First, the use of an SMS reminder before day surgery may be an efficient tool to confirm oral information given during a preanesthesia consultation. Garnier et al [14] showed that the use of SMS reminders significantly increased compliance with preoperative instructions, time of arrival, fasting rules, and hygiene rules. In our work, this decrease was associated with anesthetic causes, defined by persisting pain, postoperative nausea and vomiting, sickness, and aspiration syndrome. It is likely that an increase in compliance with preoperative instructions is associated with a decrease in intraoperative complications. Second, the use of SMS reminders may better identify subgroups of patients likely to cancel their surgery. This hypothesis is supported by the rate of cancellations observed on the day of surgery, which was higher in the SMS group compared with the telephone group (3.5% vs 2.3%). In a randomized controlled trial, Hashim et al [21] showed that the use of telephone reminders the day before an appointment significantly increased the rate of appointment cancellations, from 9.9% to 17% (*P*<.001). Moreover, our center has set up a waiting list of surgery outpatients to replace last-minute cancellation and thus avoid periods of inactivity in the operating room. Third, the use of SMS contact after surgery could improve patients’ follow-up compared with nurse telephone calls. In our study, 95.6% of patients contacted by SMS reminder after their surgery actually received the reminder. This rate seems to be higher than that found in the literature for telephone contact. In the study by Hwa and Wren [22], 78% of all outpatients were successfully contacted by telephone. According to Gaucher et al [4], this rate was 79%. With the use of SMS reminders, patients were able to respond to the reminder to report a medical problem and were then contacted by telephone by an outpatient surgery nurse. Others have demonstrated the efficiency of SMS contact on medical safety. In a study on the use of SMS reminders to follow up on colorectal surgery patients enrolled in an enhanced recovery program, Carrier et al [23] showed early alerts of postoperative complications and no missed complications.

For these reasons, we consider that the use of SMS reminders represents a safe and cost-effective method to contact patients in an outpatient surgery setting. Few studies have determined the cost and effectiveness of reminders in an outpatient surgery setting. In a meta-analysis on appointment reminders, any type of reminder, whether by SMS or telephone, increased surgery attendance rates compared with no reminder. Although similar attendance rates were found between telephone and SMS reminders, the cost of an SMS reminder was more than 2 times less than that of a telephone call [24]. One recent systematic review highlighted the usefulness of appointment reminders even if further optimization is required [25]. The impact of reminders on the health care system is not well assessed at a time when the optimization of medical time, a limited and expensive resource, is much discussed. In our work, we estimated the cost of a telephone call to be around €6 (US $6.75) including nurse time. The use of SMS reminders represents an approach that could reduce costs by one-half. Others also evaluated the social cost [26] using parameters as nonuse or the misuse of personnel time, equipment, or ward capacity in a period of shortage of resources and a political will to restrain the costs of the health care services. In a recent practice survey of French outpatient surgery units, most preoperative reminders were done by telephone by nurses (58%), and email or SMS reminders were little used (1%). Postoperative follow-up was not systematically performed but when it was done it was by telephone and mainly by nurses (70%) [3]. The use of SMS reminders instead of telephone would allow nurses to spend more time taking care of their patients on the ward and less time making time-consuming telephone calls.

### Strengths and Limitations

Our study has several limitations such as a lack of randomization. There are significant differences in patient characteristics between the groups. The 2-minute difference in the length of surgery, although statistically significant, has little overall impact. This small difference is probably related to the fact that there was more thoracic surgery than orthopedic and ear, nose, and throat surgeries. Another limitation of our study is its retrospective single-center design. However, the fact that it was conducted in an outpatient surgery unit of a university hospital center ensured the standardized organization of care teams. Our study has several strengths, including a relatively large number of patients. Also, the study period was short, which allowed us to observe the direct impact of the SMS reminders and limitations of other confounding factors.

### Conclusion

In this work, we report a decrease in the rate of conversion to full-time hospitalization with the use of pre- and postoperative SMS reminders. This new approach could represent a safe and cost-effective method in an outpatient surgery setting.

## Appendix

Multimedia Appendix 1 Examples of short message service texts in French and their translations in English.

## Abbreviations

SMS: short message service
