# Supplementary figures and images for: Efficiency of Text Message Contact on Medical Safety in Outpatient Surgery: Retrospective Study
Source: JMIR Mhealth Uhealth. 2020 Sep 10;8(9):e14346. doi: 10.2196/14346 (PMC7516679; doi:10.2196/14346)

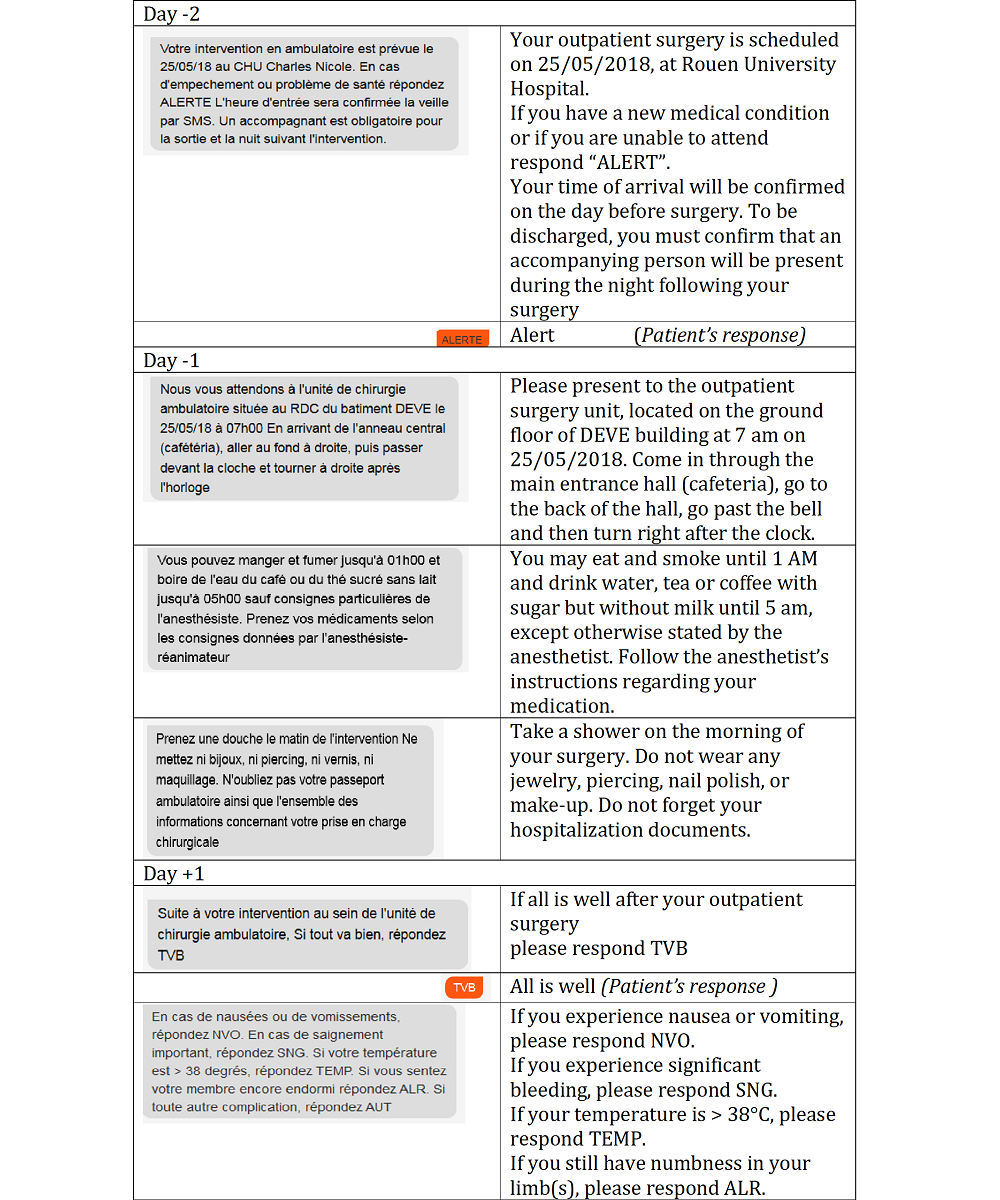

Supplement: Multimedia Appendix 1 [file mhealth_v8i9e14346_app1.png]
